# Bacterial composition of a competitive exclusion product and its correlation with product efficacy at reducing *Salmonella* in poultry

**DOI:** 10.3389/fphys.2022.1043383

**Published:** 2023-01-09

**Authors:** Margie D. Lee, Adriana A. Pedroso, John J. Maurer

**Affiliations:** ^1^ Poultry Diagnostic and Research Center, College of Veterinary Medicine, The University of Georgia, Athens, GA, United States; ^2^ Department of Biomedical Sciences and Pathobiology, Virginia Polytechnic Institute and State University, Blacksburg, VA, United States; ^3^ School of Animal Sciences, Virginia Polytechnic Institute and State University, Blacksburg, VA, United States

**Keywords:** avian, microbiome, *Salmonella*, exclusion, competition

## Abstract

The mature intestinal microbiome is a formidable barrier to pathogen colonization. Day-old chicks seeded with cecal contents of adult hens are resistant to colonization with *Salmonella*, the basis of competitive exclusion. Competitive exclusion products can include individual microbes but are commonly undefined intestinal communities taken from adult animals and in commercial production is amplified in fermentator and sold commercially in freeze dried lots. While superior to single and multiple species probiotics, reducing *Salmonella* colonization by multiple logs, undefined products have limited acceptance because of their uncharacterized status. In this study, the bacterial composition of the master stock, preproduction seed stocks and commercial lots of a poultry competitive exclusion product, was defined by 16S rRNA sequence analysis, targeting the 16S rRNA variable region (V1-V3). The samples contained a diversity of genera (22–52 distinct genera) however, the commercial lots displayed less diversity compared to the seeds and the master stock. Community composition varied between seeds and the master stock and was not a good predictor of potency, in terms of log_10_ reduction in *Salmonella* abundance. While there was significant correlation in composition between seeds and their commercial lots, this too was a not a good predictor of potency. There was linear correlation between unclassified *Actinobacteria, Peptococcus,* and unclassified Erysipelotrichaceae, and *Salmonella* abundance (*r* ^2^ > .75) for commercial seeds. However, upon review of the literature, these three genera were not consistently observed across studies or between trials that examined the correlation between intestinal community composition and *Salmonella* prevalence or abundance.

## Introduction

Many studies have shown that probiotics or direct-fed microbials can reduce intestinal disease in humans and animals (Gómez-Gallego et al., 2016; Kim et al., 2019; Lai et al., 2019; Renaud et al., 2019; Jakubczyk et al., 2020; Lucey et al., 2021; Xu et al., 2021). This is particularly important in animal production where production costs are directly impacted by intestinal health. The composition of the normal intestinal microbial community plays an important role in animal health and performance through its effect on gut morphology, nutrition, pathogenesis of intestinal disease and the immune response (Hooper et al., 2000). The term competitive exclusion (CE), introduced by Nurmi et al., 1973 is used to describe the process by which beneficial bacteria exclude *Salmonella* from the intestine (Nurmi et al., 1992).Formulations containing beneficial commensal bacteria have been marketed in some countries as probiotics, competitive exclusion formulations, or direct-fed microbials (Food and Agriculture Organization of the United Nations, 2016; Szott et al., 2022). There has been considerable interest in utilizing competitive exclusion to exclude select pathogens, especially *Salmonella*, from the gastrointestinal tract of food animals in order to reduce foodborne transmission to humans (Stern et al., 2001; Szott et al., 2022). Competitive exclusion formulations produced from the intestinal content of healthy chickens have been shown to be very effective at eliminating or reducing the prevalence of *Salmonella* in broiler chicken flocks and on carcasses (Hirn et al., 1992; Corrier et al., 1998; Stern et al., 2001).

The intestinal tract is a novel ecosystem that contains a community of bacteria rivaling the diversity of any other ecosystem on the planet. The density and number of bacteria in the community is higher than the number of host cells. Culture-based studies have suggested that intestinal microbial community is composed primarily of obligate anaerobes (Franks et al., 1998). The predominant cultivatable bacteria present in the chicken ceca are obligate anaerobes at a density of 10^11^ cells per gram of contents (Barnes et al., 1972) including at least 38 different types of anaerobic bacteria within the chicken cecum (Salanitro et al., 1974a; Barnes, 1979) with more than 200 total bacterial strains identified (Mead, 1989). However only 10%–60% of the bacteria visualized microscopically were cultured indicating a rich community of uncharacterized organisms (Barnes et al., 1972; Salanitro et al., 1974b; Barnes, 1979; Mead, 1989).

Using the DNA sequences of the small subunit ribosome genes present in a bacterial community (16S rRNA clone libraries), the composition of the intestinal community has been evaluated for many animals with very surprising results (Tannock, 1999). Commonly cultured organisms, such as *E. coli*, have been found to be a minor component of the intestine and novel uncultured organisms have been found to be the most abundant. Applications of molecular ecological profiling on poultry intestinal communities have concurred somewhat with the culture-based studies in that the chicken intestinal communities are primarily composed of Gram-positive bacteria related to mid and low G + C genera such as *Clostridia* and *Lactobacillus* (Gong et al., 2002; Lan et al., 2002; Lu et al., 2003; Zhu and Joerger, 2003). While lactobacilli are frequent in the small intestine, *Clostridia* are abundant throughout the intestinal tract of healthy chickens. The cecal intestinal microbial community is dominated by atypical and novel *Clostridia,* some of which have high G + C genomes (Apajalahti et al., 2001; Lu et al., 2003; Zhu and Joerger, 2003)*.* The DNA sequences indicate that these normal flora *Clostridia* are not closely related to pathogenic *Clostridium* (such as *perfringens*) and they do not appear to be pathogenic themselves. Only recently have the members of the order *Clostridiales* been isolated from the chicken cecum and characterized by whole genome sequencing (Medvecky et al., 2018). Approximately half of the chicken gut anaerobes (*n* = 69) were *Clostridiales*; consisting of four families (*Clostridiaceae, Erysipelotrichaceae, Lachnospiraceae*, and *Ruminococcaceae*) and, at least, 12 distinct genera (*Anaerofilum, Anaeromassillibacillus, Anaerotruncus, Blautia, Butyricicoccus, Clostridium, Drancourtella, Eubacterium, Faecalibacterium, Flavonifractor, Gemmiger*, and *Pseudoflavonifractor*). Thirty-six of these isolates show <97% or 95% 16S identity to *Clostridia* and clostridial species, respectively, in public databases and represent new clostridial genera and species awaiting taxonomic classification or reclassification. Because of their fermentative metabolism and ability to produce short chain fatty acids (SCFAs) (Medvecky et al., 2018), these organisms appear to be important players in developing an exclusive community that reduces the competition or behavior of pathogens.

To explore the genomic features of complex microbial communities, a culture-independent 16S rRNA amplicon sequencing approach has become practical and cost effective due to the advent of high throughput sequencing, a method allowing simultaneous sequencing of hundreds of thousands of individual DNA strands (Shang et al., 2018). The analysis is performed by comparing each 16S rRNA gene sequence to all others that have been detected within the community in order to determine the frequency of occurrence. The abundant organisms are frequently detected while the genes of less abundant organisms are rarely detected. However, if enough sequences are analyzed the method can reveal the presence of rare organisms. The sequence analysis can be performed at the strain level (99% DNA similarity), species level (97% DNA similarity), genus level (95% DNA similarity), and group/family level (90%). While commercial exclusion products are effective at reducing *Salmonella* colonization in poultry, their market distribution is limited by regulatory restrictions on undefined microbial products. Using a 16S rRNA-based approach, we have characterized the composition of several batches of a competitive exclusion product, marketed for controlling *Salmonella* in poultry, and used this information to determine if a particular organism or an assemblage of organisms correlated with product efficacy.

## Materials and methods

### Characterization of master seed and seed batches of a competitive exclusion product

**FIGURE 1:**
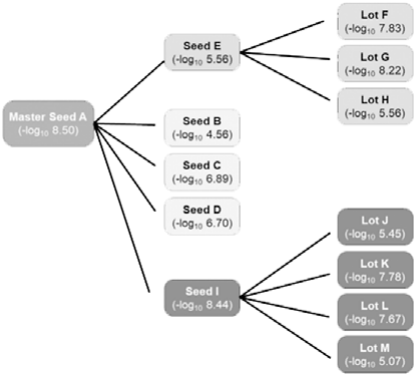
Source of competitive exclusion seeds and commercial lots. (–log10: Reduction in *Salmonella* abundance).

Commercial samples were shipped in sealed plastic bags, in the same packaging sent to customers. Thirteen samples, with potency results supplied by manufacturer were received frozen and were kept frozen until processing. All samples were opened within biosafety cabinet, previously treated with ultraviolet light and 10% bleach, and transferred to microfuge tubes for DNA extraction. Samples consisted of: master seed (sample A), two seed batches with potency <6 log_10_ reduction of *Salmonella* abundance (samples E and B), three seed batches with potency >6 log_10_ reduction (samples C, D, I), three commercial batches with potency <6 log_10_ reduction (samples H, J, M), and four commercial batches with potency >6 log_10_ reduction (samples F, G, K, L). Figure 1 shows the derivation of seeds and commercial lots from the master seed (Sample A) and their potency (-log_10_ reduction in *Salmonella* abundance). *Salmonella* reduction data was provided by the manufacturer for seeds and commercial lots; this data is used in their quality control evaluation of product efficacy. Commercial lots were released for sale if they reduced *Salmonella* colonization by at least 5 log_10_ compared to the untreated control.

### Preparation of bacterial 16S rRNA amplicons for sequencing

DNA was extracted using a MoBio Soil DNA extraction kit (MoBio Laboratories, Carlsbad California, United States) as previously described (Lu et al., 2003). The DNA quality was evaluated by gel electrophoresis with 1 kb ladder molecular weight standards. The DNA was quantified using Nanodrop (Thermo Scientific, Wilmington Delaware, United States). Barcoded PCR primers (Supplementary Tables S1, S2) targeting the 16S rRNA gene variable regions V1-V3 was used to amplify sample DNA (Hamady et al., 2008). Positive and negative, no template controls were included in the 16S PCRs, using *Salmonella* Typhimurium SR11 genomic DNA or molecular grade dH_2_O, respectively as template. All PCR reactions were set up in a PCR clean hood, in a confined room, separate from where DNA was extracted in one room and thermocyclers, housed in a physically-separate room. PCR clean hood was previously treated with ultraviolet light and surfaces were wiped down with 10% bleach. Separate set of pipettors are kept in the PCR clean hood, they are only used to set up PCR reactions, and never leave this set up area. Barrier tips are used to dispense PCR reagents and template to prevent contamination of the pipette barrels. No amplicon was observed for the negative, no template control. PCR was performed as described by Garcia et al. (2011). In order to standardize the method and to reduce PCR amplification error, 200 ng of sample DNA was used in each reaction and PCR was run for 20 cycles. Three separate PCR reactions were produced and pooled for sequencing, for each sample. Triplicate trials for each sample were done resulting in 135 independent PCR reactions. *Clostridium perfringens* ATCC 13124 DNA was used as positive control for amplification and sequencing, and a control lacking template was used to detect reagent contamination.

PCR reactions were run on agarose gels to evaluate quantity and quality of amplicon. Any sample which showed a low amount of DNA was repeated. PCR amplicons were excised from gel, purified using Qiagen Gel extraction kit (Qiagen Inc., Valencia California, United States) and pooled for each sample. Agencourt AMP pure XP kit (Beckman Coulter, Indianapolis, IN, United States) was used to further clean up and concentrate amplicon from gel extractions for 454 pyrosequencing. PCR amplicons were quantified using Nanodrop and their concentration standardized by adjusting to 10 ng/μl. Any sample which contained less than 10 ng/μl was repeated in order to produce the necessary amount of DNA for standardization. Samples were submitted to the Georgia Genomics and Bioinformatics Core (University of Georgia, Athens, GA, United States) for pyrosequencing using 454 protocols established by Roche Inc. (Branford Connecticut, United States) which manufactures the instrument and reagents.

### Bioinformatics and statistical analyses

The sequence analysis pipeline was performed using Roche and Mothur software (Schloss et al., 2009). Sequences were sorted by barcodes in order to organize by sample, trimmed based on size (≤500 bp) and quality to remove those sequences with gaps or ambiguous base calls. Sequences were aligned in order to detect and remove chimeras produced from PCR artifact.

The bacterial diversity was calculated comparing the similarity of the sequences and those deposited at Ribosomal Project Database (http://rdp.cme.msu.edu/) using the RDP6 database. The bacterial composition based on genus (95% similarity) and family (90% similarity) was generated in order to determine the phylogenetic composition of each sample. Sørensen’s coefficient of similarity (QS) was used to quantify the similarity of samples (Sorenson, 1948). QS = 2C/A + B where A and B are the number of species in samples A and B, respectively, and C is the number of species shared by the two samples. Sørensen’s coefficient ranges from 0–1. Simple correlation was used to statistically verify the similarity of sample composition. Chi-squared test was used in order to test differences in the proportion of the genus observed among the samples. Linear regression was adopted to determine the correlation between the bacterial genus and the levels of *Salmonella* reduction reported for commercial lots or seeds. Analysis of covariance was used to identify the effect of the seed on the commercial product. Statistic tests were performed using SAS software.

### Screening samples for *Salmonella*

γ-Proteobacterial 16S sequences do not exhibit enough sequence diversity to reliably identify *Salmonella* and differentiate it from closely related member species. Therefore, some sequences may be erroneously reported as genus: *Salmonella* by Mothur, using the RDP6 database. In order to determine if *Salmonella* was present in commercial samples, a diagnostic PCR, targeting a *Salmonella*-specific locus, was applied. Fifty ng of sample DNA was used in PCR reactions, as described by Liu et al., 2002 (Liu et al., 2002) using *invA* primers. The samples were screened by gel electrophoresis using a 1.5% agarose gel containing ethidium bromide in order to visually detect amplicons of the expected size (450 bp). *Salmonella* Typhimurium genomic DNA served as a positive amplification control. A no template control was included to identify PCR contamination (false positive). The no template control was consistently negative in these *Salmonella* PCR screens.

## Results

### Sequencing quality control and culling anomalous sequences, ambiguities and chimeras from final sequence dataset

725,293 total sequences were obtained from the pyrosequencing reactions (Supplementary Table S3). However, after elimination of anomalous long sequences, homopolymers, and ambiguous bases, 703,522 sequences were subjected to chimera analysis for additional quality control. Chimeras are PCR artifacts that erroneously increase sample diversity and alter composition (Ley et al., 2008). After these quality control procedures, 332,559 sequences were of sufficient quality for compositional and statistical analysis. The distribution of sequences among the samples varied from 1,913–64,816 (Supplementary Table S3). The reads were therefore normalized to run data analysis for correlation, chi-square test, linear regression and covariance.

### 16S-rRNA based compositional analysis of CE seeds and commercial lots

**FIGURE 2:**
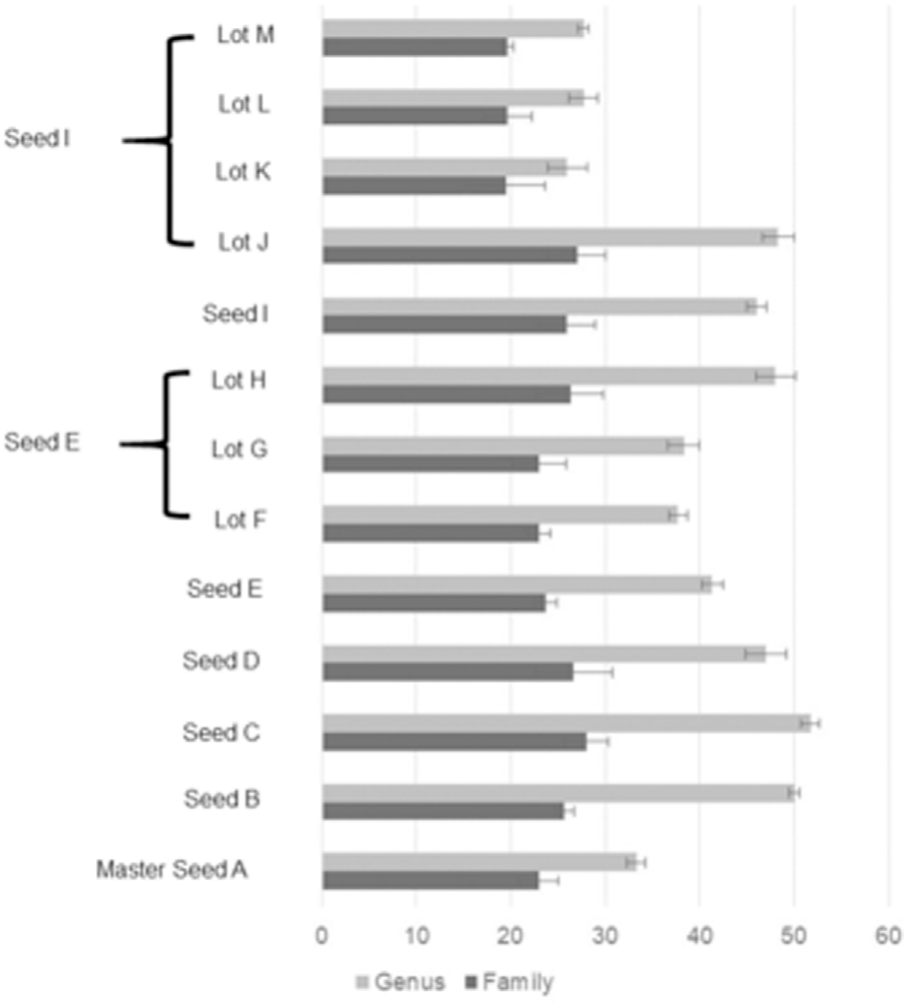
Number of different taxa in 16S rRNA sequences from master seed A, seed stocks (B**–**E, I) and commercial lots (F**–**H, J**–**M) at family (90% similarity) and genus (95% similarity) level. The error bars estimate sequence analysis error detected by sequencing the same sample three times.

**FIGURE 3:**
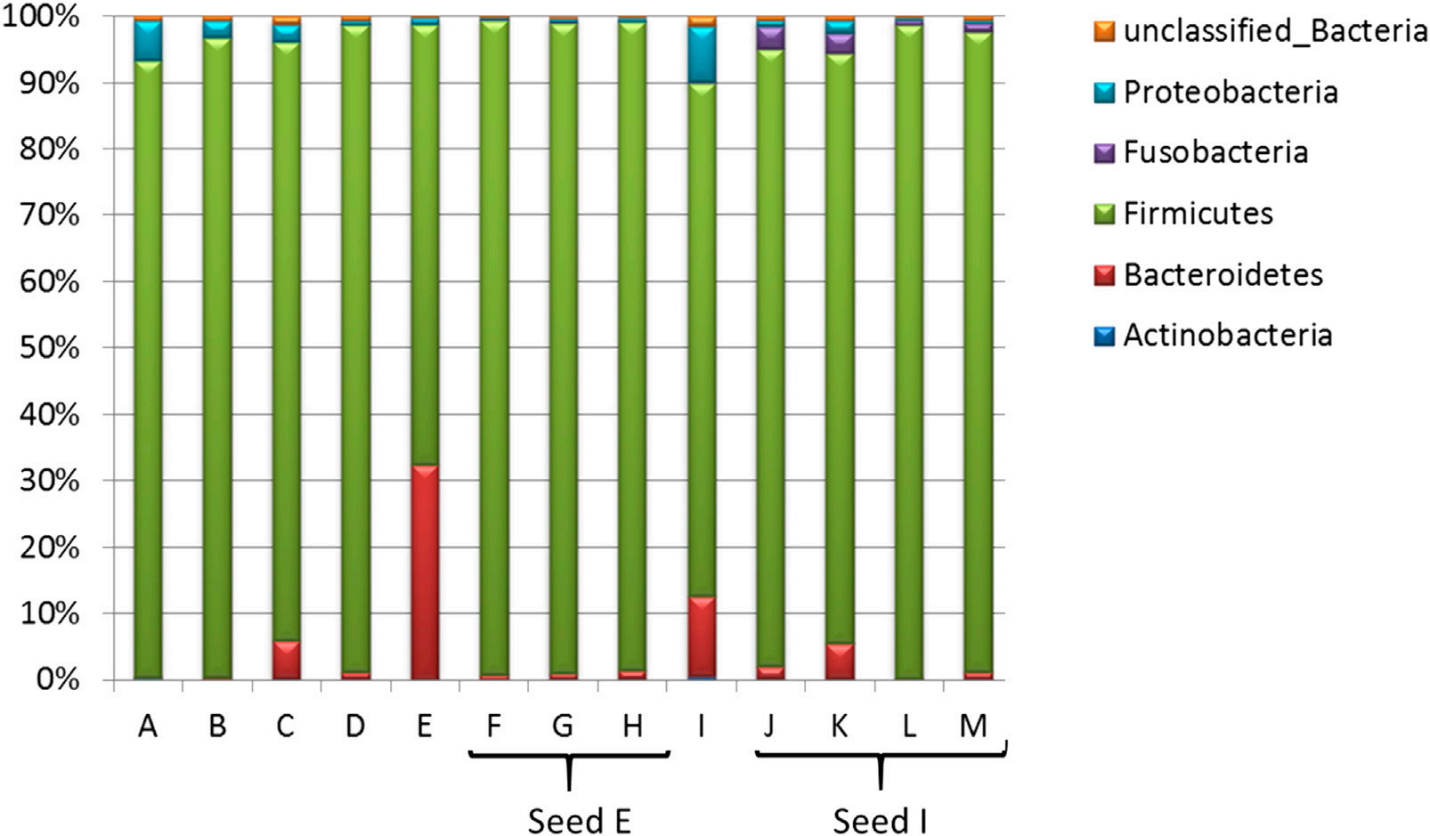
Bacterial composition of competitive exclusion master seed, seed stocks and derived commercial lots at the phyla level.

**FIGURE 4:**
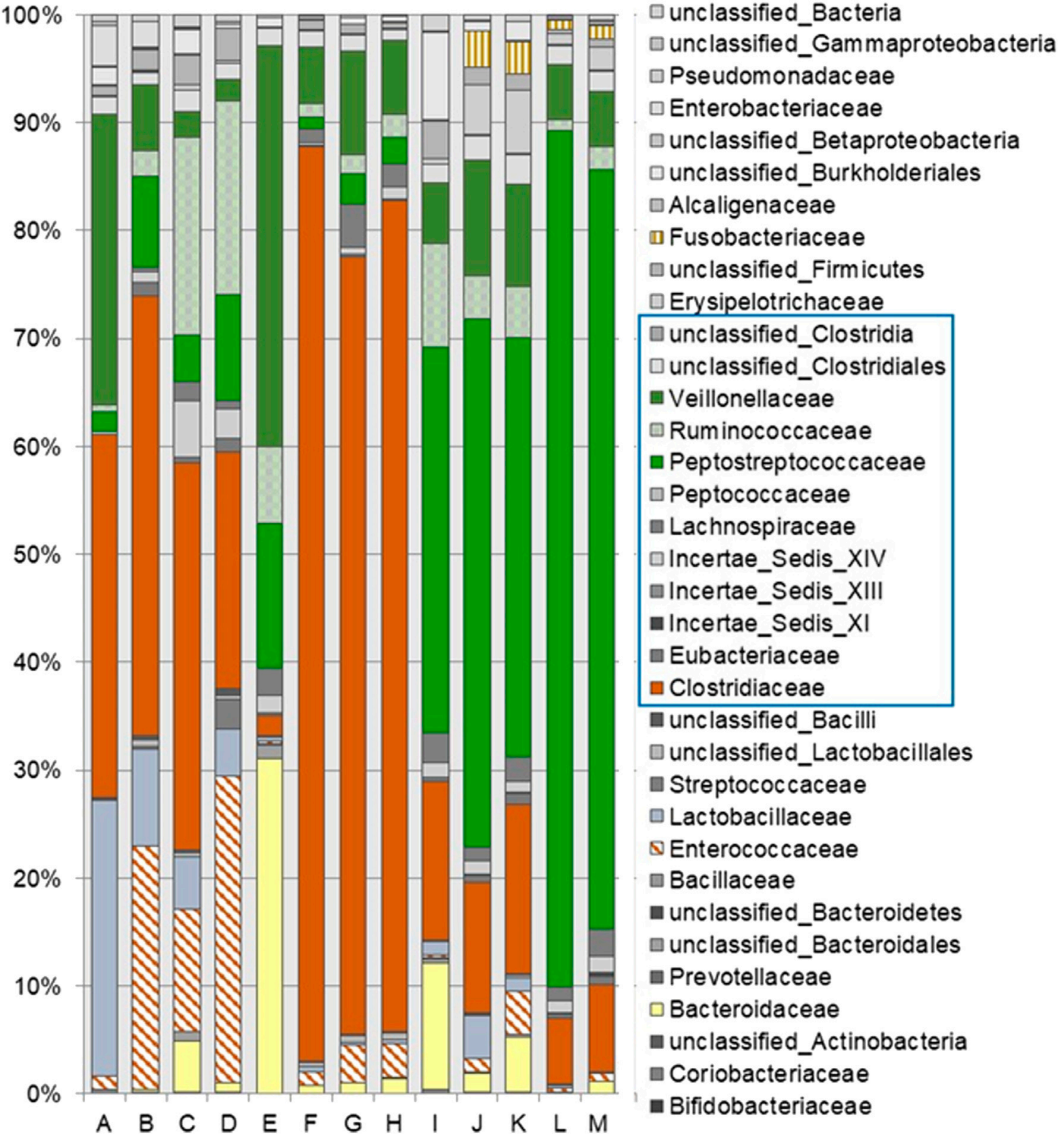
Bacterial composition of competitive exclusion master seed, seed stocks and derived commercial lots at the family level. The most abundant organisms are shown in **bold**, families belonging to *Clostridia* are denoted by the blue box.

The number of families detected varied from 15 to 28 and the number of genera from 22 to 52 for each sample (Figure 2). The seeds tended to contain the largest number of genera (mean = 45) with commercial lots containing the fewest (mean = 34). There was no correlation between the number of genera or families with *Salmonella* reduction. The bacterial composition is presented at the phyla (Figure 3), family (Figure 4; Table 1) and genus (Table 2) level. Unclassified bacteria, organisms that have yet been assigned a phylum, made up the smallest proportion, while *Firmicutes* was the dominant phylum, throughout CE samples, with the order *Clostridia* representing the most abundant group (58%–95.5%) within the phylum Firmicutes. Twelve different taxonomic families of *Clostridia* were detected in CE product, indicating that there was high diversity within this order. Firmicutes and the minor proteobacteria were the two phyla present in the master seed. Other phyla were identified in seeds and commercial lots, varying in their proportion, and included phyla Bacteroidetes, Fusobacteria, and Actinobacteria. While CE seed E had the highest proportion of Bacteroidetes (>30%), the proportion of this phyla in commercial lots derived from this seed was low. The same was observed for CE seed I, with regards to the 2nd major phyla, Proteobacteria (∼10%) and abundance of this phyla in resulting commercial lots were sporadic and low. *Clostridiaceae*, *Veillonellaceae*, and *Lactobacillaceae*, were the major families present in the master seed; their proportion in seeds and commercial lots varied. The proportion of these three families in the seeds was not predictive of their abundance in the resulting, commercial lots. Bacteria belonging to the families *Clostridiaceae, Bacteroidaceae, Lactobacillaceae, Enterococcaceae, Peptostreptococcaceae,* and *Veillonellaceae* comprised more than 20% of any one sample. The seeds tended to contain higher proportions of *Lactobacillaceae* and *Enterococcaceae* while the commercial lots tended to have higher levels of *Peptostreptococcaceae*. Seed I, which was a progenitor seed stock for commercial lots J–M had a highest proportion of *Peptostreptococcaceae*, of the seed stocks. This seed stock proved the most efficacious of the seeds at reducing *Salmonella*. The resulting commercial lots, generated from this seed stock, also had a significantly high proportion of this bacterial family.

**TABLE 1:** Proportions of bacterial orders or families detected in competitive exclusion seeds and commercial lots.

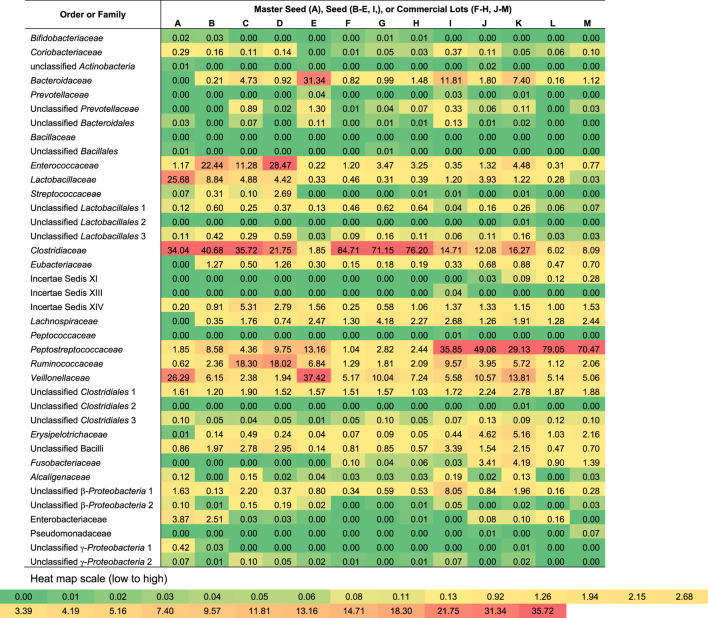

**TABLE 2:** Proportion of genera detected in competitive exclusion seeds and commercial lots.

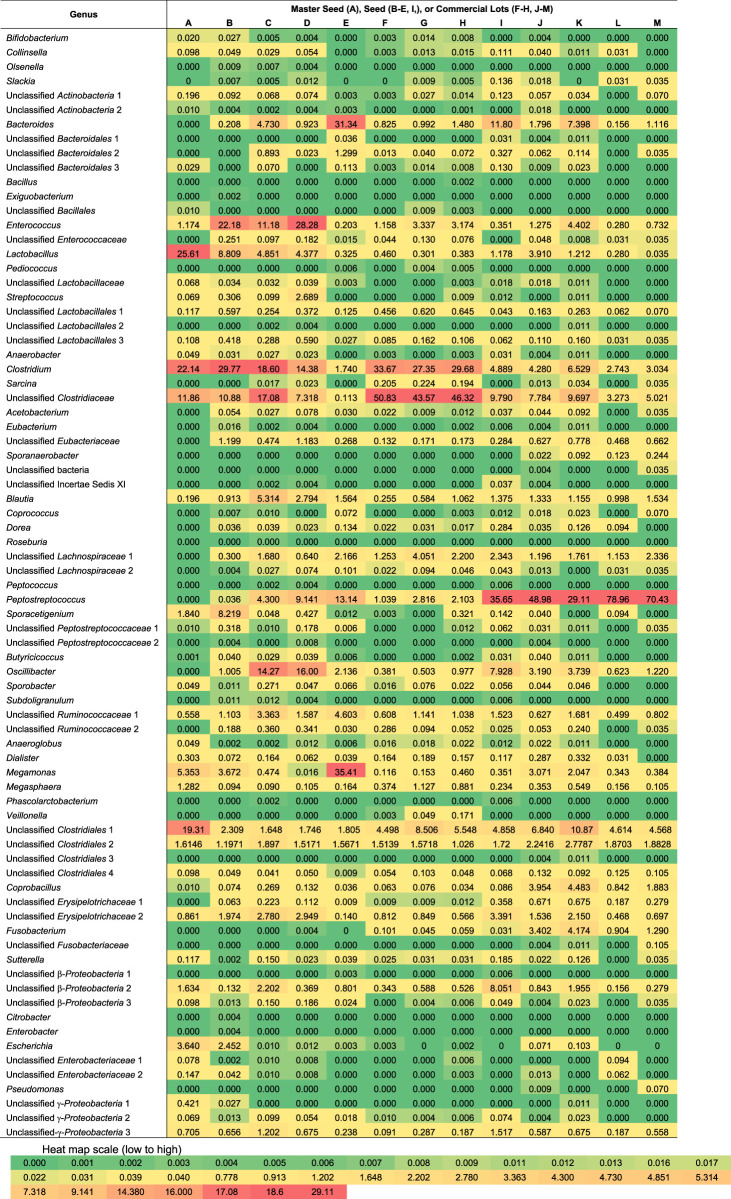

γ-Proteobacteria were detected among all of the samples and in a number of samples the Mothur software reported *Salmonella* among the genera detected. γ-Proteobacterial 16S sequences, and particularly the *Enterobacteriaceae* family, do not exhibit enough sequence diversity to be reliably used to report *Salmonella* because other genera within this group have highly similar 16S sequences*.* A diagnostic PCR was used to screen commercial samples for *Salmonella*. This PCR test has been used extensively to screen clinical and environmental samples for the presence of *Salmonella* (Liu et al., 2002). None of the samples gave a positive reaction therefore the erroneously classified sequences were corrected in the report to reflect their identity as an unknown *Enterobacteriaceae* species.

### Consistency in bacterial composition of commercial lots with their seeds is not a good predictor of product efficacy

Table 3 presents the similarity between the community composition of the master seed, at the family and genus level, and the seeds produced from it. Statistical analysis, using simple correlation, varied across seeds compared to the master (Family: .719–.272; Genus .641–.078). Similarity with the master seed was not a good predictor of efficacy as the seed most similar to the master and with a high correlation value (Seed B) had the lowest *Salmonella* log_10_ reduction compared to one of the power seeds, with regards to community similarity (Seed I; Table 3). While individual seeds produced commercial lots with similar community composition at the family and genus level, this was not a good predictor as to which lots would be expected to have the greatest reduction of *Salmonella* counts (Table 4). Even comparing commercial lots against themselves, varied in their ability to reduce *Salmonella* abundance, was not a significant predictor of product efficacy. However, community similarity tended to reflect commercial lots origins with seed stocks used to generate CE product (Table 5).

**TABLE 3:** Similarity in bacterial composition of the master seed A with seeds B, C, D, E, or I.

Seeds	Log_10_ reduction^a^	Family similarity coefficient	Family correlation^b^ ^,^ ^c^	Genus similarity coefficient	Genus correlation^1^ ^,^ ^2^
B	−4.56	.893	.719	.750	.641
C	−6.89	.847	.624	.727	.541
D	−6.70	.678	.390	.722	.320
E	−5.56	.630	.360	.721	.078
I	−8.44	.750	.272	.696	.153

^a^ Reduction in *Salmonella* abundance.

^b^ Pearson’s correlation coefficient.

^c^ Not significant.

**TABLE 4:** Similarity in bacterial composition between seeds, exhibiting high (Seed I) or low (Seed E) *Salmonella* reduction, with their commercial lots.

Seeds	Log_10_ reduction^a^	Family similarity coefficient	Family correlation^b,c^	Genus similarity coefficient	Genus correlation^1^ ^,^ ^2^
I	−8.44				
J	−5.45	.857	.916	.796	.922
K	−7.78	.885	.921	.849	.933
L	−7.67	.784	.873	.690	.889
M	−5.07	.852	.888	.731	.901
E	−5.56				
F	−7.83	.889	.038	.835	−.001
G	−8.22	.873	.103	.826	.013
H	−5.56	.881	.073	.857	.017

^a^ Reduction in *Salmonella* abundance.

^b^ Pearson’s correlation coefficient.

^c^ Not significant.

**TABLE 5:** Similarity in the bacterial composition of commercial lots produced from seeds E (F–H) and I (J–M) as defined by Sørensen’s coefficient and simple correlation ( ).

Family	F (−7.83)^a^	G (−8^.^22)^a^	H (−5.56)^a^	J (−5.45)^a^	K (−7.78)^a^	L (−7.67)^a^	M (−5.07)^a^
Genus							
F (−7.83)^a^		.945 (.995)	.915 (.999)	.885 (.215)	.847 (.431)	.857 (.058)	.746 (.094)
G (−8.22)^a^	0.899 (.992)		.933 (.998)	.871 (.250)	.833 (.478)	.800 (.085)	.868 (.122)
H (−5.56)^a^	.843 (.998)	.854 (.997)		.879 (.238)	.844 (.460)	.778 (.077)	.807 (.114)
J (−5.45)^a^	.808 (.169)	.781 (.213)	.864 (.193)		.879 (.926)	.786 (.970)	.847 (.978)
K (−7.78)^a^	.825 (.340)	.796 (.400)	.847 (.371)	.885 (.936)		.815 (.824)	.842 (.845)
L (−7.67)^a^	.769 (.050)	.759 (.090)	.717 (.071)	.702 (.980)	.667 (.870)		.894 (.999)
M (−5.07)^a^	.660 (.079)	.800 (.119)	.735 (.100)	.800 (.986)	.774 (.886)	.757 (.999)	

^a^ −log_10__: Reduction in *Salmonella* abundance. Family comparison is highlighted in light gray. *p*-values for Pearson’s correlation coefficient were >.05.

### Identification of genera within seeds and commercial lots that correlated with reduction of *Salmonella* colonization in chickens

**FIGURE 5:**
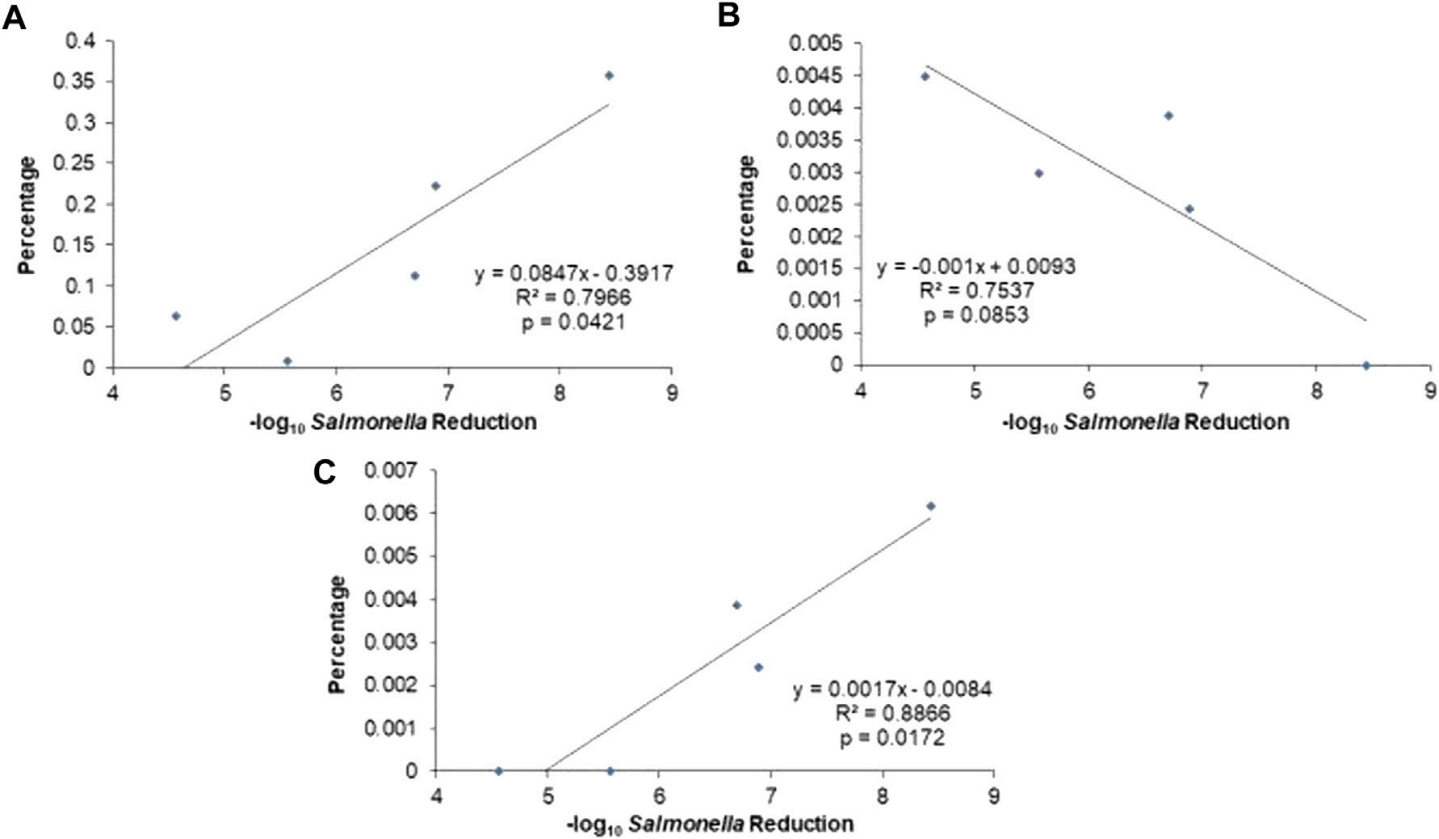
Linear correlation between genus abundance and −log10 reduction in *Salmonella* by competitive exclusion seeds B, C, D, E, and I. **(A)** Unclassified *Erysipelotrichaceae 1*. **(B)** Unclassified *Actinobacteria 2*. **(C)** *Peptococcus*. *Salmonella* reduction data was provided by the manufacturer for seeds and commercial lots.

Linear regression was performed to determine if a particular organism correlated with *Salmonella* reduction. In the seeds, unclassified *Actinobacteria, Peptococcus,* and unclassified *Erysipelotrichaceae* correlated with product efficacy (log_10_ *Salmonella* reduction) at *r* ^2^ greater than 75% (Table 6, Supplementary Tables S4, S5). There was a linear correlation among these organisms with *Salmonella* reduction in the seeds (Figure 5); specifically, an antagonistic relationship for unclassified *Erysipelotrichaceae* and *Peptococcus versus* a facultative relationship with *Actinobacteria*. In contrast, linear regression did not detect an organism correlating significantly with efficacy in the commercial lots themselves (Supplementary Table S5), including the afore mentioned genera. However, because all of the commercial products produced at least a 5 log_10_ reduction in *Salmonella* colonization, it may be difficult to detect correlation using this method. Furthermore, *Salmonella* reduction may not be due to the actions of a particular organism but due to the combined metabolic activity of organisms which produce an exclusive community.

**TABLE 6:** Genera shared with the master competitive exclusion stock with seeds as determined by chi-square test.

Genera	Master seed A (−log_10_ 8.50) vs.	Master seed A (−log_10_ 8.50) vs.	Master seed A (−log_10_ 8.50) vs.	Master seed A (−log_10_ 8.50) vs.	Master seed A (−log_10_ 8.50) vs.
	Seed B (−log_10_ 4.56)	Seed E (−log_10_ 5.56)	Seed D (−log_10_ 6.70)	Seed C (−log_10_ 6.89)	Seed I (−log_10_ 8.44)
*Bacteroides*	ns	.001	ns	.03	.001
*Enterococcus*	.001	ns	.001	.001	ns
*Lactobacillus*	ns	.001	.001	.001	.001
*Anaerobacter*	ns	.001	ns	ns	.001
*Sarcina*	ns	.001	ns	ns	.001
unclassified *Clostridiaceae*	.002	ns	ns	ns	ns
*Blautia*	ns	ns	ns	.03	ns
*Peptostreptococcus*	ns	.001	.001	ns	.001
*Megamonas*	ns	.001	.02	.04	.04
*Veillonella*	.001	.001	.001	.001	.001

−log_10_: Reduction in *Salmonella* abundance. Ns-not significant. Not statistically significant association of genus (*n* = 68), in comparison of seed stocks with the master: *Bifidobacterium*; *Collinsella*; *Olsenella*; *Slackia*; unclassified *Actinobacteria* 1,2; unclassified *Bacteroidales* 1–3; *Bacillus*; *Exiguobacterium*; unclassified *Bacillales*; unclassified *Enterococcaceae*; *Pediococcus*; unclassified *Lactobacillaceae*; *Streptococcus*; unclassified *Lactobacillales* 1–3; *Clostridium*; *Acetobacterium*; *Eubacterium*; unclassified *Eubacteriaceae*; *Sporanaerobacter*; unclassified bacteria; unclassified Incertae Sedis XI; *Coprococcus*; *Dorea*; *Roseburia*; unclassified *Lachnospiraceae* 1,2; *Peptococcus*; *Sporacetigenium*; unclassified *Peptostreptococcaceae* 1,2; *Butyricicoccus*; *Oscillibacter*; *Sporobacter*; *Subdoligranulum*; unclassified *Ruminococcaceae* 1,2; *Anaeroglobus*; *Dialister*; *Megasphaera*; *Phascolarctobacterium*; unclassified *Clostridiales* 1–4; *Coprobacillus*; unclassified *Erysipelotrichaceae* 1,2; *Fusobacterium*; unclassified *Fusobacteriaceae*; *Sutterella*; unclassified β-*Proteobacteria* 1–3; *Citrobacter*; *Enterobacter*; *Escherichia*; unclassified *Enterobacteriaceae* 1,2; *Pseudomonas*; unclassified γ-*Proteobacteria* 1–3.

## Discussion

The host must maintain surveillance over the composition of the microbiota and exhibit control over the abundance and behavior of members that affect the host-microbe homeostasis. Therefore transfer of microbial communities from parent to offspring may have evolved to initially establish ecological health within the lumen of host mucosal systems and reduce susceptibility to mucosal disease (Neish, 2009). A number of studies have demonstrated that administration of complex microbial communities can reduce the ability of *Salmonella* to colonize young animals; these have been extensively reviewed (Nurmi et al., 1992; Nisbet, 1998; Becker, 2005) including those specifically addressing poultry production (Ferreira et al., 2003). Complex microbial communities, such as those used in competitive exclusion, have been shown in multiple independent studies to be effective in reducing *Salmonella* in poultry (Cameron et al., 1996; Cameron et al., 1997; Deruyttere et al., 1997; Guillot et al., 1997; Guerra-Garcia Miranda, 2000; Stephan, 2000; Sisak et al., 2001; Nakamura et al., 2002; Ferreira et al., 2003). Products that consist of single bacteria, such as *Lactobacillus* and *Bacillus*, are less efficacious (Nurmi et al., 1992). However the complex microbial communities are difficult to characterize using classical bacteriological methods (Hume et al., 1996).

The competitive exclusion product contains a diversity of microbial species, many of which are not consistently present in preproduction seeds or commercial lots. In fact, there are some taxa shared between seeds or lots that were not detected in the master by our methods. *Peptostreptococcus*, abundant in commercial lots, was not detected in the Master Seed, but had to be present to seed these lots and suggests limits of this 16S rRNA gene sequencing approach to detect minor taxa. While certain taxa were identified that seemed to correlate with *Salmonella* abundance, they are not consistently observed across studies (Azcarate-Peril et al., 2018; Ma et al., 2020; Leyva-Diaz et al., 2021; Pedroso et al., 2021). While others have identified a similar correlation between prevalence or abundance of certain taxa with *Salmonella* colonization in poultry, these taxa are also not uniformly present in other studies or even between trials, in the same study (Videnska et al., 2013; Azcarate-Peril et al., 2018; Ma et al., 2020; Mon et al., 2020; Pedroso et al., 2021). The bacterial composition of this competitive exclusion product was not reflected in the cecal community composition in animals administered the commercial product (Pedroso et al., 2016). This may be due, in part, to the microbial succession that occurs in the maturation of the intestinal microbiome (Lu et al., 2003). However, the CE product does contain members that appear in an intestinal compartment, ileum or cecum, at some point in the intestine’s development (Lu et al., 2003). Community diversity appears to be key in pathogen exclusion (Pedroso et al., 2021) in that it is not as important who is present as what the community does “collectively” to exclude *Salmonella*. Others have also noted the importance of community diversity in pathogen exclusion (van Elsas et al., 2012; Antharam et al., 2013; Lone et al., 2013; Stanley et al., 2014; Xu et al., 2014; Zhang et al., 2015; Chopyk et al., 2016). This diversity provides resilience needed to return to homeostasis following some perturbation to the system (Weimer, 2015). As long as there are sufficient members present that collectively perform a specific function, the system is maintained; in this case microbiome’s ability to maintain a barrier to pathogen colonization. Therefore, seeding the chick with the adult intestinal microbiome, provides it with sufficient members, collectively capable of excluding *Salmonella* while its own microbiome develops as the animal matures.

It is unclear how the products exclude pathogens but it seems unlikely that composition is only one characteristic responsible for their protective effects. The possible mechanisms of action of competitive exclusion formulations have been previously reviewed (Nurmi et al., 1992; Nisbet, 1998; Tuomola et al., 2001; Becker, 2005). Bacteria comprising exclusive communities may: produce bactericidal molecules that damage the cellular integrity of pathogenic bacteria; decrease the growth rate of pathogens by providing competition for nutrients or produce molecules that inhibit processes involved in cell division; produce molecules that reduce expression of, or function of, factors involved in colonization; cause or enhance predation; or physically occupy or modify the ecological niche targeted by the pathogen. Multiple mechanisms are likely responsible for pathogen exclusion. This would explain why diversity is key; why no one specie(s) was consistently associated with *Salmonella* exclusion in this study. *Salmonella* is metabolically versatile in that it can utilize some metabolites (ethanolamine, propanediol, etc.) that few other community members can. A metabolic gene(s) involved in competition for substrate A may be distributed across a diversity of bacterial species, where any one, member species could compete for substrate A. This may explain why no one bacterial species has been consistently associated with reduced *Salmonella* abundance. While an intestinal member species may be able to compete with *Salmonella* for one substrate or metabolite, *Salmonella* could turn to another, and another substrate enabling persistent colonization even with low energy substrates. Only organisms with similar metabolic potential could outcompete *Salmonella*, especially another *Salmonella* (Cheng et al., 2015). Therefore, if competition were the mechanism of competitive exclusion, it would take a broad array of member species for the community to outcompete *Salmonella* for all the substrates and metabolites present in the different portions of the intestine.

Similarly, antagonism may be at the heart of competitive exclusion. The antibacterial activity of a competitive bacterial species may be attributed to several factors (Alakomi et al., 2005). For example, the mechanism of antibacterial activity of exclusive lactobacilli might be due to a synergistic action of lactic acid and bacteriocins. Lactate acts as a permeabilizer of the outer membrane of Gram-negative bacteria, increasing their susceptibility to antimicrobial molecules (Fayol-Messaoudi et al., 2005). Lactate also affects the intestinal pH which may affect the surface structures and metabolism of *Salmonella* (Foster et al., 1994)*.* Other bacterial metabolites, such as acetate, propionate and butyrate, may also contribute to community exclusion of some bacterial species because the undissociated organic acids freely diffuses across the bacterial membrane, lowering the cytoplasmic pH and uncoupling electron transport (Ricke, 2003). The intestinal tract contains high levels of these volatile short chain fatty acids (SCFA) which are produced from the breakdown of complex carbohydrates by anaerobes such as the *Clostridia, Bacteroides,* and *Bifidobacterium*. SCFAs have inhibitory effects on *Salmonella* colonization of the gastrointestinal tract (Bohnhoff et al., 1964); and can modulate expression of *Salmonella* invasion genes (Durant et al., 1999; Durant et al., 2000a; Durant et al., 2000b). In addition, bile salt deconjugation by *Clostridia*, to form cholate and deoxycholate, can also synergistically inhibit *Salmonella* invasion (Ducarmon et al., 2019). The culmination of all of these factors are likely at play in pathogen exclusion requiring collective species metabolic activity.

Community diversity appears to be important in providing an ecosystem with multi-functionality and redundancy of function (Delgado-Baquerizo et al., 2016a; Delgado-Baquerizo et al., 2016b). Diversity maintains function, even when a perturbation is introduced (Isobe et al., 2020). Most juveniles obtain their microbiomes from the parent (Ferretti et al., 2018; Kubasova et al., 2019; Zhu et al., 2021; Chen et al., 2022). The adult microbiome provides the young with pioneer colonizers important in intestinal development, immune function and pathogen exclusion. In poultry production, juveniles are separated from the adult hen, prior to hatch. Seeding chicks with a competitive exclusion product provides an effective barrier to pathogen colonization, that would be otherwise absent in this production environment.

## Conclusion

Pathogen exclusion may be a combination of competition and antagonism. In either case, a bacterial census is not likely to identify the magic bullet, the single organism, responsible for excluding *Salmonella* from a mixed community as exists in the chicken intestine. The key to understanding competitive exclusion will come from comparing communities that permit and exclude *Salmonella* and associated transcriptome, proteome, or metabolome evidence of competition and antagonism (García et al., 2017). Regulatory agencies are starting to come around to commercial acceptance of these fecal communities for treating or preventing bacterial infections, as evident from US Food and Drug Administration’s approval of fecal microbiota product for the treatment of *Clostridium difficile* infections (Pharmaceutical Technology Editors, 2022).

## Data Availability

16S sequence data is available through MG-RAST, metagenomics analysis server (mg-rast.org). The individual data sets can be accessed through https://www.mg-rast.org/linkin.cgi?project = followed by mgp40703 (Commercial Lot M), mgp40704 (Commercial Lot L), mgp4075 (Commercial Lot K), mgp40706 (Commercial Lot J), mgp40707 (Competitive Exclusion Seed I), mgp40708 (Commercial Lot H), mgp40709 (Commercial Lot G), mgp40710 (Commercial Lot F), mgp40711 (Competitive Exclusion Seed E), mgp40712 (Competitive Exclusion Seed D), mgp40713 (Competitive Exclusion Seed C), mgp40714 (Competitive Exclusion B) and mgp40715 (Competitive Exclusion Master Seed A). Therefore, to access Master Seed A, the link is https://www.mg-rast.org/linkin.cgi?project=mgp40715.
