# Supplementary material for: Bacterial composition of a competitive exclusion product and its correlation with product efficacy at reducing Salmonella in poultry
Source: Front Physiol. 2023 Jan 9;13:1043383. doi: 10.3389/fphys.2022.1043383 (PMC9868637; doi:10.3389/fphys.2022.1043383)
Supplement: Supplementary file 1 [file Table1.pdf]

Table S1. Forward PCR primers targeting V1-V3 region of 16S rRNA gene

Name	Sequence
TiBac-B-27F-YM	CCTATCCCCTGTGTGCCTTGGCAGTCTCAGAGAGTTTGATYMTGGCTCAG
TiBac-B-27F-Bif	CCTATCCCCTGTGTGCCTTGGCAGTCTCAGAGGGTTCGATTCTGGCTCAG
TiBac-B-27F-Bor	CCTATCCCCTGTGTGCCTTGGCAGTCTCAGAGAGTTTGATCCTGGCTTAG
TiBac-B-27F-Chl	CCTATCCCCTGTGTGCCTTGGCAGTCTCAGAGAATTTGATCTTGGTTCAG

Nucleotide symbols: Y = C or T; M = A or C

Table S2. Reverse barcoded PCR primers targeting V1-V3 region of 16S rRNA gene

Name	Sequence
TiBac-A-P1-R	CCATCTCATCCCTGCGTGTCTCCGACTCAGAACCGCATCCGCNGCKGCTGGCAC
TiBac-A-P2-R	CCATCTCATCCCTGCGTGTCTCCGACTCAGAACCGCTACCGCNGCKGCTGGCAC
TiBac-A-P3-R	CCATCTCATCCCTGCGTGTCTCCGACTCAGAACCTTCCCCGCNGCKGCTGGCAC
TiBac-A-P4-R	CCATCTCATCCCTGCGTGTCTCCGACTCAGAAGGAACCCCGCNGCKGCTGGCAC
TiBac-A-P5-R	CCATCTCATCCCTGCGTGTCTCCGACTCAGAAGGTTCCCCGCNGCKGCTGGCAC
TiBac-A-P6-R	CCATCTCATCCCTGCGTGTCTCCGACTCAGACACACACCCGCNGCKGCTGGCAC
TiBac-A-P7-R	CCATCTCATCCCTGCGTGTCTCCGACTCAGACACCTCTCCGCNGCKGCTGGCAC
TiBac-A-P8-R	CCATCTCATCCCTGCGTGTCTCCGACTCAGACACCTGACCGCNGCKGCTGGCAC
TiBac-A-P9-R	CCATCTCATCCCTGCGTGTCTCCGACTCAGACACGACTCCGCNGCKGCTGGCAC
TiBac-A-P10-R	CCATCTCATCCCTGCGTGTCTCCGACTCAGACACGAGACCGCNGCKGCTGGCAC
TiBac-A-P11-R	CCATCTCATCCCTGCGTGTCTCCGACTCAGACACTCTCCCGCNGCKGCTGGCAC
TiBac-A-P12-R	CCATCTCATCCCTGCGTGTCTCCGACTCAGACACTGACCCGCNGCKGCTGGCAC
TiBac-A-P13-R	CCATCTCATCCCTGCGTGTCTCCGACTCAGACCTACCTCCGCNGCKGCTGGCAC
TiBac-A-P14-R	CCATCTCATCCCTGCGTGTCTCCGACTCAGACCTACGACCGCNGCKGCTGGCAC
TiBac-A-P15-R	CCATCTCATCCCTGCGTGTCTCCGACTCAGACCTCATCCCGCNGCKGCTGGCAC
TiBac-A-P16-R	CCATCTCATCCCTGCGTGTCTCCGACTCAGACCTCTACCCGCNGCKGCTGGCAC
TiBac-A-P17-R	CCATCTCATCCCTGCGTGTCTCCGACTCAGACCTGAACCCGCNGCKGCTGGCAC
TiBac-A-P18-R	CCATCTCATCCCTGCGTGTCTCCGACTCAGACCTGTTCCCGCNGCKGCTGGCAC
TiBac-A-P19-R	CCATCTCATCCCTGCGTGTCTCCGACTCAGACCTTCGTCCGCNGCKGCTGGCAC
TiBac-A-P20-R	CCATCTCATCCCTGCGTGTCTCCGACTCAGACCTTGGACCGCNGCKGCTGGCAC
TiBac-A-P21-R	CCATCTCATCCCTGCGTGTCTCCGACTCAGACGAACCTCCGCNGCKGCTGGCAC
TiBac-A-P22-R	CCATCTCATCCCTGCGTGTCTCCGACTCAGACGAACGACCGCNGCKGCTGGCAC
TiBac-A-P23-R	CCATCTCATCCCTGCGTGTCTCCGACTCAGACGAAGGTCCGCNGCKGCTGGCAC
TiBac-A-P24-R	CCATCTCATCCCTGCGTGTCTCCGACTCAGACGACATCCCGCNGCKGCTGGCAC
TiBac-A-P25-R	CCATCTCATCCCTGCGTGTCTCCGACTCAGACGACTACCCGCNGCKGCTGGCAC
TiBac-A-P26-R	CCATCTCATCCCTGCGTGTCTCCGACTCAGACGAGAACCCGCNGCKGCTGGCAC
TiBac-A-P27-R	CCATCTCATCCCTGCGTGTCTCCGACTCAGACGAGTTCCCGCNGCKGCTGGCAC
TiBac-A-P28-R	CCATCTCATCCCTGCGTGTCTCCGACTCAGACGATGGACCGCNGCKGCTGGCAC
TiBac-A-P29-R	CCATCTCATCCCTGCGTGTCTCCGACTCAGACTCACTCCCGCNGCKGCTGGCAC
TiBac-A-P30-R	CCATCTCATCCCTGCGTGTCTCCGACTCAGACTCCTCACCGCNGCKGCTGGCAC
TiBac-A-P31-R	CCATCTCATCCCTGCGTGTCTCCGACTCAGACTCCTGTCCGCNGCKGCTGGCAC
TiBac-A-P32-R	CCATCTCATCCCTGCGTGTCTCCGACTCAGACTCGTCTCCGCNGCKGCTGGCAC
TiBac-A-P33-R	CCATCTCATCCCTGCGTGTCTCCGACTCAGACTCTCACCCGCNGCKGCTGGCAC
TiBac-A-P34-R	CCATCTCATCCCTGCGTGTCTCCGACTCAGACTCTGTCCCGCNGCKGCTGGCAC
TiBac-A-P35-R	CCATCTCATCCCTGCGTGTCTCCGACTCAGACTGACACCCGCNGCKGCTGGCAC
TiBac-A-P36-R	CCATCTCATCCCTGCGTGTCTCCGACTCAGACTGGAGACCGCNGCKGCTGGCAC
TiBac-A-P37-R	CCATCTCATCCCTGCGTGTCTCCGACTCAGACTGTCTCCCGCNGCKGCTGGCAC
TiBac-A-P38-R	CCATCTCATCCCTGCGTGTCTCCGACTCAGAGACACTCCCGCNGCKGCTGGCAC
TiBac-A-P39-R	CCATCTCATCCCTGCGTGTCTCCGACTCAGAGACCTCACCGCNGCKGCTGGCAC

TiBac-A-P40-R	CCATCTCATCCCTGCGTGTCTCCGACTCAGAGACGACACCGCNGCKGCTGGCAC
TiBac-A-P41-R	CCATCTCATCCCTGCGTGTCTCCGACTCAGAGACTCACCCGCNGCKGCTGGCAC
TiBac-A-P42-R	CCATCTCATCCCTGCGTGTCTCCGACTCAGAGAGACACCCGCNGCKGCTGGCAC
TiBac-A-P43-R	CCATCTCATCCCTGCGTGTCTCCGACTCAGAGAGAGTCCCGCNGCKGCTGGCAC
TiBac-A-P44-R	CCATCTCATCCCTGCGTGTCTCCGACTCAGAGAGGAGACCGCNGCKGCTGGCAC
TiBac-A-P45-R	CCATCTCATCCCTGCGTGTCTCCGACTCAGAGGAAGGACCGCNGCKGCTGGCAC
TiBac-A-P46-R	CCATCTCATCCCTGCGTGTCTCCGACTCAGAGGACAACCCGCNGCKGCTGGCAC

Nucleotide symbols: N = any nucleotide; K = T or G

Table S3. Quality filtering of 16S rRNA sequences (n = 725,293): elimination of anomalous long reads, homopolymers, ambiguous bases and chimeric sequences.

Sample	Number of sequences[1]	Number of sequences[2]
A	18,325	10,219
B	99,731	44,691
C	101,288	41,329
D	66,624	25,773
E	76,353	33,566
F	50,739	31,772
G	39,650	22,268
H	112,298	64,816
I	41,594	16,221
J	51,000	22,662
K	22,254	8,745
L	7,233	3,208
M	6,742	2,868
N	5,559	2,508
O	4,132	1,913
Total	703,522	332,559

[1]Elimination of anomalous long reads, homopolymers, and ambiguous bases resulting from sequencing errors. [2]Elimination of chimeric sequences following filter for anomalous long reads, homopolymers and ambiguous bases.

Table S4. Genera shared among seeds as determined by chi-square test.

Genera	B vs E	B vs C	B vs D	B vs I	C vs E	C vs D	C vs I	D vs I
Bacteroides[1]	0.001	0.04	ns	0.001	0.001	ns	ns	0.001
Enterococcus[1]	0.001	ns	ns	0.001	0.001	0.01	0.001	0.001
Clostridium[2]	0.001	ns	0.02	0.001	0.001	ns	0.001	0.03
Unclassified *Clostridiaceae*[1]	ns	ns	ns	ns	0.001	0.05	ns	ns
Peptostreptococcus[1]	0.001	0.04	0.001	0.001	ns	ns	0.001	0.001
Sporacetigenium[2]	0.001	0.001	0.01	0.01	ns	ns	ns	ns
Oscillibacter[2]	ns	0.001	0001	0.02	0.001	ns	ns	ns
Megamonas[1]	0.001	ns	ns	ns	0.001	ns	ns	ns
unclassified β-*Proteobacteria* 2[2]	ns	ns	ns	ns	0.01	ns	ns	0.01

ns-not significant. No statistically significant association of genus (n = 69) among seeds: *Bifidobacterium*; *Collinsella*; *Olsenella*; *Slackia*; unclassified *Actinobacteria* 1,2; unclassified *Bacteroidales* 1-3; *Bacillus*; *Exiguobacterium*; unclassified *Bacillales*; unclassified *Enterococcaceae*;

***Lactobacillus*[1]**; *Pediococcus*; unclassified *Lactobacillaceae*; *Streptococcus*; unclassified *Lactobacillales* 1-3; ***Anaerobacter*[1]; *Sarcina*[1];** *Acetobacterium*; *Eubacterium*; unclassified *Eubacteriaceae*; *Sporanaerobacter*; unclassified bacteria; unclassified Incertae Sedis XI; ***Blautia*[1];** *Coprococcus*; *Dorea*; *Roseburia*; unclassified *Lachnospiraceae* 1,2; *Peptococcus*; unclassified *Peptostreptococcaceae* 1,2; *Butyricicoccus*; *Subdoligranulum*; unclassified *Ruminococcaceae* 1,2; *Anaeroglobus*; *Dialister*; *Megasphaera*; *Phascolarctobacterium*; ***Veillonella*[1];** unclassified *Clostridiales* 1-4; *Coprobacillus*; unclassified *Erysipelotrichaceae* 1,2; *Fusobacterium*; unclassified *Fusobacteriaceae*; *Sutterella*; unclassified β-*Proteobacteria* 1,3; *Citrobacter*; *Enterobacter*; *Escherichia*; unclassified *Enterobacteriaceae* 1,2; *Pseudomonas*; unclassified γ-*Proteobacteria* 1-3. [1]Statistically significant association identified in comparison of the master stock with seeds. [2]No statistically significant association of genus in comparison of the master stock with seeds.

Table S5. Genera shared with progenitor seeds E and I and among their commercial lots as determined by chi-square test.

	Seeds	Commercial Lots[1]								
Genus	E vs I	F vs G	F vs H	G vs H	K vs L	J vs M	J vs K	K vs M	L vs J	L vs M
Bacteroides[2]	0.003	ns	ns	ns	0.01	ns	ns	0.03	ns	ns
Lactobacillus[2]	ns	ns	ns	ns	ns	0.05	ns	ns	ns	ns
unclassified *Clostridiaceae*[2]	0.002	ns	ns	ns	ns	ns	ns	ns	ns	ns
Peptostreptococcus[2]	0.001	ns	ns	ns	0.0001	ns	0.02	0.001	0.01	ns
Megamonas[2]	0.0001	ns	ns	ns	ns	ns	ns	ns	ns	ns
Unclassified β-*Proteobacteria* 3	0.015	ns	ns	ns	ns	ns	ns	ns	ns	ns

ns-not significant. Not statistically significant association of genus (n = 69), in comparison between seeds E and I and among commercial lots F-H, J-M derived from these two seeds: *Bifidobacterium*; *Collinsella*; *Olsenella*; *Slackia*; unclassified *Actinobacteria* 1,2; unclassified *Bacteroidales* 1-3; *Bacillus*; *Exiguobacterium*; unclassified *Bacillales*; ***Enterococcus***[2]; unclassified *Enterococcaceae*; *Pediococcus*; unclassified *Lactobacillaceae*; *Streptococcus*; unclassified *Lactobacillales* 1-3; ***Anaerobacter***[2]**;** *Clostridium*; ***Sarcina***[2]**;** *Acetobacterium*; *Eubacterium*; unclassified *Eubacteriaceae*;

Sporanaerobacter; unclassified bacteria; unclassified Incertae Sedis XI; *Coprococcus*; *Dorea*; *Roseburia*; unclassified *Lachnospiraceae* 1,2; *Peptococcus*; *Sporacetigenium*; unclassified *Peptostreptococcaceae* 1,2; *Butyricicoccus*; *Oscillibacter*; *Sporobacter*; *Subdoligranulum*; unclassified *Ruminococcaceae* 1,2; *Anaeroglobus*; *Dialister*; *Megasphaera*; *Phascolarctobacterium*; ***Veillonella*[2]*;** unclassified *Clostridiales* 1-4; *Coprobacillus*; unclassified *Erysipelotrichaceae* 1,2; *Fusobacterium*; unclassified *Fusobacteriaceae*; *Sutterella*; unclassified β-*Proteobacteria* 1,2; *Citrobacter*; *Enterobacter*; *Escherichia*; unclassified *Enterobacteriaceae* 1,2; *Pseudomonas*; unclassified γ-*Proteobacteria* 1-3. [1]Seed E is the progenitor stock for commercial lots F-H. Seed I is the progenitor stock for commercial lots J-M. [2]Statistically significant association identified in comparison of the master stock with seeds.
